# Fount, fate, features, and function of renal erythropoietin-producing cells

**DOI:** 10.1007/s00424-022-02714-7

**Published:** 2022-06-24

**Authors:** Sophie L. Dahl, Andreas M. Bapst, Stellor Nlandu Khodo, Carsten C. Scholz, Roland H. Wenger

**Affiliations:** 1 Institute of Physiology and National Centre of Competence in Research “Kidney.CH”, University of Zürich, CH-8057 Zurich, Switzerland; 2 Institute of Physiology, University Medicine Greifswald, D-17475 Greifswald, Germany

**Keywords:** Erythropoietin, Fibrosis, Hypoxia, Kidney, Oxygen sensing, Protein hydroxylation

## Abstract

Renal erythropoietin (Epo)-producing (REP) cells represent a rare and incompletely understood cell type. REP cells are fibroblast-like cells located in close proximity to blood vessels and tubules of the corticomedullary border region. Epo mRNA in REP cells is produced in a pronounced “on–off” mode, showing transient transcriptional bursts upon exposure to hypoxia. In contrast to “ordinary” fibroblasts, REP cells do not proliferate ex vivo, cease to produce Epo, and lose their identity following immortalization and prolonged in vitro culture, consistent with the loss of Epo production following REP cell proliferation during tissue remodelling in chronic kidney disease. Because Epo protein is usually not detectable in kidney tissue, and Epo mRNA is only transiently induced under hypoxic conditions, transgenic mouse models have been developed to permanently label REP cell precursors, active Epo producers, and inactive descendants. Future single-cell analyses of the renal stromal compartment will identify novel characteristic markers of tagged REP cells, which will provide novel insights into the regulation of Epo expression in this unique cell type.

## Erythropoietin function or functions?

The only known essential function of the endocrine glycoprotein hormone erythropoietin (Epo) is the regulation of red blood cell (RBC) homeostasis. Epo dysregulation inevitably leads to disturbances of RBC production in the bone marrow [118]. Mouse models with targeted deletions of the genes encoding Epo or the Epo receptor (EpoR) are non-viable due to lethal anemia during embryonic development [121], whereas transgenic Epo overexpression causes massive erythrocytosis [84]. The finding of Epo expression in cells that are separated from the blood stream by a tight barrier, such as in brain and testis, sparked the idea of local paracrine/autocrine Epo functions beyond erythropoiesis. Indeed, a myriad of non-erythropoietic Epo functions have been suggested [118]. However, these functions have commonly been investigated by injections of unphysiologically high doses of recombinant Epo, in the absence of appropriate controls such as inactive/mutant recombinant Epo produced in a blinded manner along with wild-type Epo and diluted in the same stabilization solution. Non-erythropoietic Epo functions should also be confirmed in EpoR knockout mice containing an erythroid-specific transgenic EpoR rescue [109]. At least under disease conditions, non-erythropoietic variants of Epo seem to be tissue protective via an alternative heterodimeric EpoR [11], but the physiological relevance of these findings remains questionable.

While during fetal life the liver is the main Epo-producing organ, in adult mammals the kidney is essential for endocrine Epo production [33, 87]. Patients with end-stage renal disease (ESRD) usually require Epo injections to maintain their RBC counts, and there appears to be no other additional Epo function that depends on these treatments. This review will hence focus on the (dys)regulation of Epo production in the kidney and the implications for the treatment of renal anemia in ESRD.

## Renal Epo regulation: much lauded but still incompletely understood

**Fig. 1:**
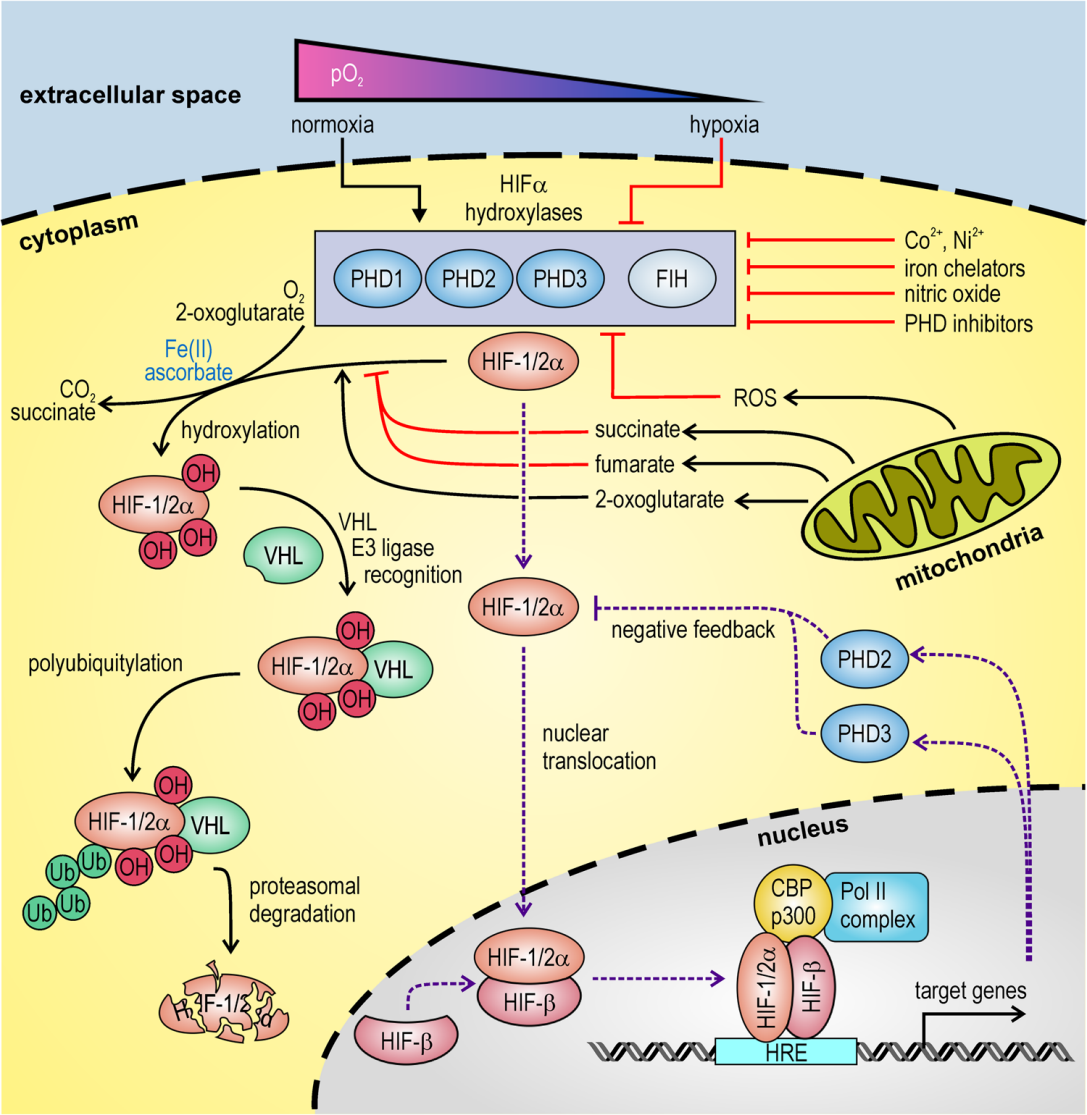
Oxygen sensing and signalling. The prolyl-4-hydroxylase domain (PHD) enzymes PHD1, PHD2, and PHD3, and the asparaginyl hydroxylase factor inhibiting HIF (FIH) utilize the co-substrates molecular oxygen and 2-oxoglutarate (2-OG) to hydroxylate the hypoxia-inducible factor (HIF) α subunits, along with the conversion of 2-OG to succinate by oxidative decarboxylation. Ferrous iron and reducing agents such as ascorbate (vitamin C) serve as co-factors required for enzymatic function. Hydroxylase activity is inhibited by hypoxia, several Krebs cycle intermediates and agents that interfere with ferrous iron, including transition metals, iron chelators, nitric oxide, and other oxidative reactive oxygen species (ROS). Hydroxylated HIFα is recognized by the von Hippel-Lindau (VHL) ubiquitin E3 ligase adaptor protein, and subsequently subjected to proteasomal degradation. Non-hydroxylated HIFα heterodimerizes with the common HIF-β subunit and forms a transcriptional enhancer complex at hypoxia response elements (HREs) of HIF target genes. PHD2 and PHD3 are among these genes, establishing a negative feedback loop that limits HIF activity and adapts the hypoxic set point to the microenvironmental oxygen partial pressure

Somewhat unusual for an endocrine hormone, the rate-limiting step in renal Epo synthesis takes place on the level of gene expression. Tissue hypoxia rather than “RBC counting” induces Epo transcription, explaining the identical Epo response following hypoxemia and anemia. Transgenic mouse models identified the regulatory regions in the distal 5′ and proximal 3′ *EPO* gene flanking regions which direct *EPO* gene expression in the kidney and liver, respectively [56, 67, 68, 90, 91, 93]. Detailed analysis of the 3′ flanking region led to the discovery of the hypoxia-inducible factor (HIF) family of transcription factors [92, 94]. In 2019, the elucidation of the HIF signalling cascade was honored by the bestowal of the Nobel Prize in Physiology or Medicine (previously reviewed in detail in this journal [28]). As outlined in Fig. 1, the α subunits of these heterodimeric transcription factors are oxygen-labile, marked for degradation by oxygen-dependent prolyl-4-hydroxylases (PHDs) and conveyed to the ubiquitin–proteasome system by the von Hippel-Lindau (VHL) tumor suppressor protein [89]. Factor inhibiting HIF (FIH), another HIF hydroxylase, is apparently not involved in Epo regulation [114, 124]. Like the collagen hydroxylases, HIFα hydroxylases are sensitive to oxidation and require ascorbate (vitamin C) as a co-factor. However, we and others have shown that alternative reducing agents (glutathione, dithiothreitol) can replace vitamin C in vitro [31, 73], and we subsequently confirmed that vitamin C deficiency in *Gulo* knockout mice does not lead to Epo overproduction in vivo [73].

The molecular mechanisms governing renal *EPO* gene expression are well established: under hypoxic conditions, HIF-2α is stabilized and forms together with HIF-β the transcription factor HIF-2 that binds *cis*-regulatory elements of the *EPO* gene to enhance its transcription [86, 116]. However, this mechanism has primarily been investigated using hepatoma cell culture models and/or the liver-specific 3′ flanking region. The absence of a suitable kidney-derived cell culture model, and the finding that the 3′ flanking region is dispensable for kidney-restricted *EPO* gene expression [107], limited the understanding of kidney-specific Epo production, which in the adult is physiologically more relevant than liver-specific Epo production. Kidney cells capable of Epo synthesis are generally referred to as renal Epo-producing (REP) cells and include currently active cells as well as cells bearing the ability to produce Epo.

Open questions include the molecular identity of these REP cells, from origin to fate; the precise interplay of the various tissue-specific *cis*-enhancer elements governing the organ/cell type restriction and hypoxia-inducibility of *EPO* gene expression; the burst-like “on–off” transcription pattern, shutting down *EPO* expression even under ongoing hypoxic conditions; the unique oxygen sensitivity in vivo; the route of the instantaneous endocrine Epo secretion; the sex-independent levels of circulating Epo despite sex-specific differences in blood hemoglobin content; the mechanism of the loss of Epo production during ESRD; and the apparent specificity of the pan-PHD inhibitors for Epo induction, in healthy as well as in diseased kidneys.

## Genetics to provide more insights into Epo regulation?

Knockout mouse models confirmed the essential functions of the genes encoding HIF-2α (*EPAS1*), PHD2 (*EGLN1*), and VHL (*VHL*) in the regulation of *EPO* gene expression [44, 116]. Other members of the HIFα and PHD families may have modulatory functions, but their precise roles in REP cell biology, if any, are unknown. Conditionally targeted mouse models are of limited help because REP cells are rare and specific marker genes, other than *Epo* itself, which would be suitable to drive REP-specific Cre expression, are currently unknown. Moreover, Cre drivers with REP cell overlapping expression patterns used for conditional knockouts of VHL and PHDs cause unphysiologically high, constitutive, and isoform-independent HIFα stabilization, often leading to ectopic Epo production [13, 32, 37, 38, 55, 61, 62].

Patients suffering from congenital erythrocytosis as well as populations living over evolutionary relevant periods in high altitude provided additional insights into the genetics of Epo regulation. Mutations in the genes encoding HIF-2α (but not HIF-1α and HIF-3α), PHD2 (but not PHD1 and PHD3), and VHL have been identified to be associated either with congenital secondary (i.e., Epo-dependent) erythrocytosis or with blunted erythropoietic responses, typical for high-altitude adaptation of RBC homeostasis [6, 8, 64, 66, 105]. Furthermore, some rare mutations in the *EPO* gene itself cause erythrocytosis [112, 125]. Regarding the fact that the majority of all congenital erythrocytosis cases are of idiopathic origin, it appears likely that this approach will lead to novel candidate loci involved in Epo regulation [16]. However, it will be a major challenge to unequivocally elucidate the direct mechanistic connection between these loci and renal Epo expression. Because the expression of most of these factors is widespread, non-renal functions, e.g., in steroid hormone-producing organs or in the bone marrow, must be considered as well [81].

Genome-wide association studies (GWAS) linking single-nucleotide polymorphisms (SNPs) with RBC traits (i.e., hemoglobin content, hematocrit, and RBC size and counts) provided new insights into RBC dysregulation and have the potential to identify new candidate regulators of Epo production. Genes involved in iron metabolism, proliferation of erythropoietic precursor cells, or the *EPO* locus itself have been identified [3, 17, 19, 36, 59, 97, 113]. However, there is much less information about SNPs associated with circulating Epo levels, probably because of the high costs and strong variability of circulating Epo which, among others, shows very low basal levels and fluctuates in a circadian manner. One study focused on ageing populations with gradually decreasing renal function [9]. Some smaller studies looked at Epo after kidney transplantation [39, 40], or at the (lack of) sex-specific differences in chronically anemic patients [51]. None of these initial analyses has been performed in a larger cohort of randomly chosen, healthy individuals of the normal population.

The first reported Epo-GWAS study in normal individuals identified a single intergenic locus between the *HBS1L* and *MYB* genes [42]. This locus had previously been found to be associated with deregulated fetal hemoglobin (HbF) in a background of a Chinese β-thalassemia anemia population [29, 101], which is likely permissive for the selection of otherwise erythrocytosis-causing mutations. Disruption of this locus in the mouse prevented binding of several erythropoietic transcription factors, lowered *Myb* gene activation, and increased HbF synthesis [106]. Using dried neonatal blood spots, also the *CRP* locus was reported to be associated with circulating Epo [115]. In contrast to the latter study, we found a strong heritability of circulating Epo levels in a family-based cohort from the general population [21]. SNPs of the *MAP2K5*-*SKOR1*-*PIAS1* locus showed the highest association with circulating Epo levels. However, to date the direct mechanistic links between the *HBS1L*-*MYB*, *CRP*, and *MAP2K5*-*SKOR1*-*PIAS1* loci and Epo remain unknown.

## Regulatory elements conferring tissue specificity and hypoxia inducibility to *EPO* gene expression

As outlined above, mouse models containing transgenic DNA fragments of the human *EPO* locus allowed for the identification of tissue-specific and hypoxia-inducible *cis*-acting DNA regions, but the resolution of this approach was not sufficient to make any conclusions about the involved transcription factor-binding sites. Using hepatoma cell lines [41], the ~ 50-bp *EPO* 3′ hypoxia response element (HRE) has extensively been characterized [92, 94]. This HRE turned out to be active in basically all the cultured cell types [70], and similar functional HREs can be found in hundreds of HIF target genes involved in hypoxia adaptation [119]. However, the mouse *Epo* 3′ HRE is not required for hypoxia-inducible Epo expression in the kidney [107], and we suggested that a distal 5′ HRE is involved, instead [104].

**Fig. 2:**
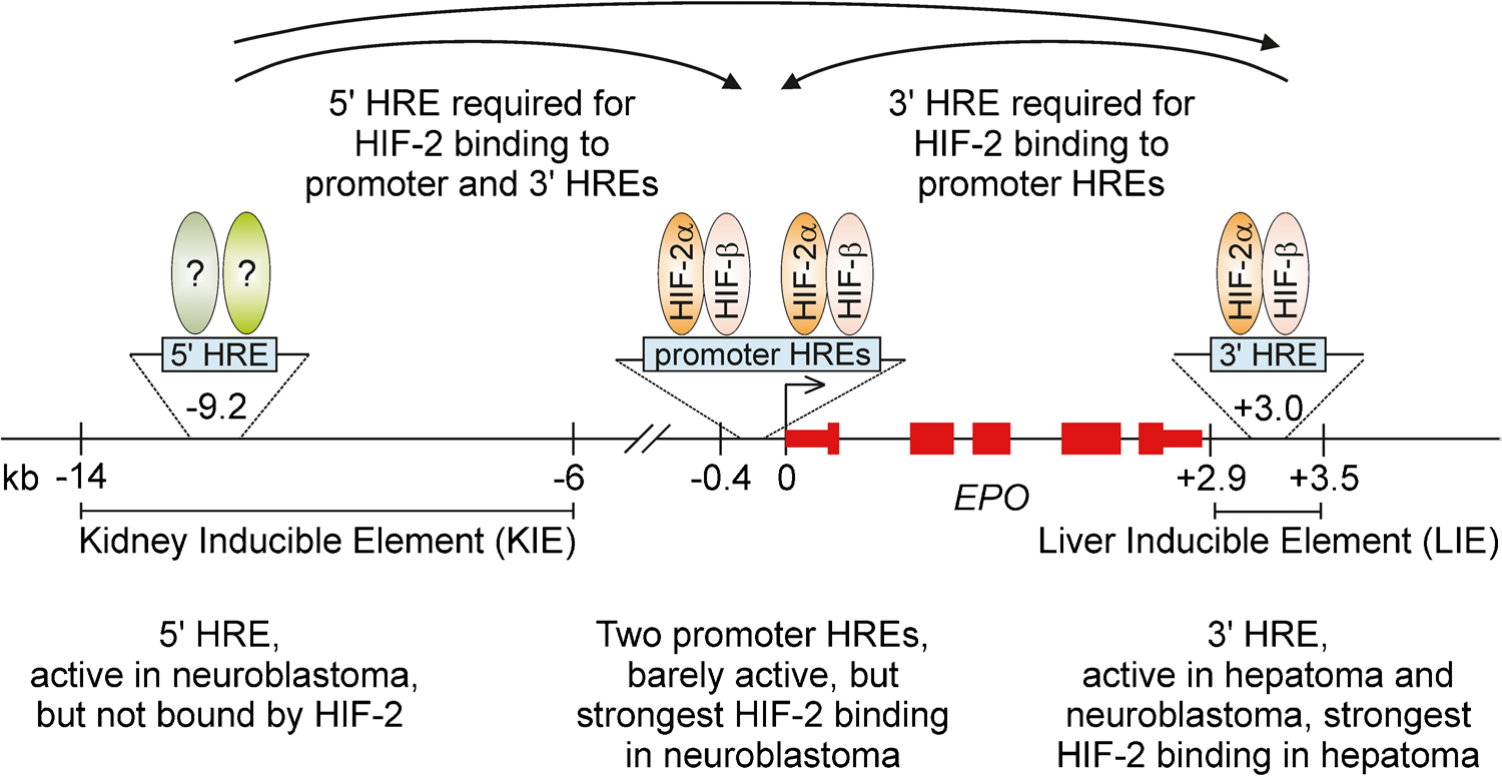
Regulatory elements of the human *EPO* gene. Hypoxia response elements (HREs) have been identified in the distal 5′ and 3′ enhancer regions as well as in the proximal promoter region of the *EPO* gene. Hypoxia-inducible transcription factor complexes interact with these HREs in a cell-type–specific manner to govern *EPO* gene expression as indicated. kb, kilobases

Because of the transient nature of Epo expression in REP cells cultivated ex vivo, to date no renal cell culture model has been used for the analysis of putative kidney regulatory *EPO cis*-elements. Regarding the expression of several neuronal lineage markers in REP cells in vivo [2, 75], it seemed likely that in human Kelly neuroblastoma cells, known to express Epo in a hypoxia-inducible manner [103], *EPO cis*-regulatory elements may be involved which are not active in hepatoma cells. Indeed, using CRISPR-Cas9-mediated mutational analyses, only the 3′ HRE was found to be required in Hep3B hepatoma cells, but both the 5′ and 3′ HREs contribute to the hypoxic induction of endogenous Epo mRNA in Kelly cells [77]. Chromatin immunoprecipitation (ChIP) experiments confirmed the preferential interaction of HIF-2 (and to some extent HIF-1) with the 3′ HRE in hepatoma cells. Despite its essential function in neuroblastoma cells, neither HIF-2 nor HIF-1 bound the 5′ HRE, but there was an unexpected strong HIF interaction with two newly identified HREs in the promoter region, although the minimal *EPO* promoter alone showed only a weak hypoxia-inducible activity. Intriguingly, mutation of either the distal 5′ or 3′ endogenous HREs completely abolished HIF binding to the proximal *EPO* promoter HREs as well as to the 3′ HRE in neuroblastoma cells [77]. Figure 2 summarizes the differences between human hepatic and neuronal cells in the complex remote interaction of *cis*-regulatory DNA elements and *trans*-acting protein factors of the *EPO* gene locus.

Using mouse embryonic fibroblast (MEF)-derived Epo-producing C3H10T1/2 cells, the interaction of HIF-2 (and HIF-1) with the HREs located in the 5′, promoter, and 3′ regions of the *Epo* gene has recently been confirmed by ChIP [96]. This finding was unexpected since MEFs usually do not express any HIF-2α and there is no remaining HIF-2 response in HIF-1α-deficient MEFs [102]. The factors governing Epo expression in this particular MEF-derived cell line hence remain to be investigated.

That each of these three HRE-containing elements of the *EPO* locus confers hypoxia inducibility to a reporter gene has previously been shown in Hep3B hepatoma cells, but the same authors disputed the necessity of the 5′ HRE for hypoxic induction of Epo in the mouse kidney [47]. Therefore, they phlebotomized transgenic mice containing a large bacterial artificial chromosome (BAC) construct of the mouse *Epo* locus with a 0.7-kb deletion of the 5′ HRE region and GFP in place of most of the *Epo* exons. While GFP remained undetectable in normal mice, strong GFP signals were still observed in the kidney of anemic mice [47]. Because the hypoxia-inducible *Epo* promoter (as well as the 3′ HRE) was still present in this transgene, the conclusion that the putative 5′ HRE is not active in REP cells seems not to be justified. While it may well be that only neuronal but not renal Epo regulation requires the specific 5′ HRE sequence within the crucial upstream regulatory region, our Kelly experiments demonstrated an unprecedented remote action of both distal 5′ and 3′ HREs on HIF occupancy of the promoter HREs at least in neuronal cells, which may also inspire future experiments to elucidate renal Epo regulation, possibly involving a currently unknown additional kidney-specific 5′ HRE.

## The enigmatic nature of REP cells: targeting the unknown

The identity of the renal cells that produce Epo has been debated for decades, mainly due to the limited quality of the original in situ hybridization (ISH) technology [108]. With the development of much more specific and sensitive ISH techniques (the most widely applied method known as “RNAscope”), it is now undisputed that only fibroblast-like cells located in the intertubular space, often referred to as “interstitial” cells, produce Epo mRNA. These peritubular and pericytic REP cells are mainly located in the corticomedullary border region but can be found throughout the cortex in severe anemia [58, 69, 122].

**Fig. 3:**
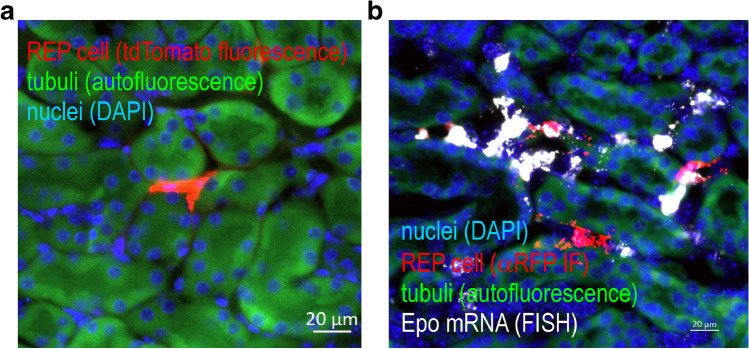
Renal Epo-producing (REP) cells. *Epo-Cre*^*ERT2*^x*tdTomato* reporter mice were treated with tamoxifen and exposed for 4 h to 0.1% carbon monoxide (CO) resulting in ~ 50% CO saturation of hemoglobin (hypoxemia), a strong but short-lived stimulus for Epo expression. **a** Fluorescence microscopy of a REP cell 3 weeks after the permanent labelling with fluorescent tdTomato protein (red). **b** Reporter mice were treated with a second identical hypoxic stimulus 1 week after the initial REP tagging, and analyzed immediately by Epo mRNA fluorescent in situ hybridization (FISH; white). Because the FISH procedure destroyed its fluorescence, tdTomato protein was detected by anti-RFP immunofluorescence (αRFP IF; red). **a**, **b** Tubuli were visualized by their autofluorescence (green) and nuclei were stained by 4′,6-diamidino-2-phenylindole (DAPI; blue)

Because (i) REP cells are rare, (ii) normoxic Epo protein is basically undetectable, and (iii) hypoxic Epo mRNA expression occurs only transiently; independent markers to target and analyze these cells are urgently required. A number of Cre drivers have been used to target REP cells, including promoter elements derived from the genes encoding CD68, renin, connexin 40, PDGFRβ, and FOXD1 [13, 32, 37, 38, 54, 55, 61, 62]. Because these genes are also expressed in non-REP cells, the corresponding models are probably of limited use to study physiological Epo regulation. The only reliable alternative to tag REP cells is the use of the *Epo* gene itself. However, in contrast to the mentioned Cre drivers, a small promoter fragment of the *Epo* gene is not sufficient to recapitulate tissue-specific Epo expression. Alternatively, a reporter gene could be inserted into the endogenous *Epo* locus by homologous recombination, but this led to *Epo* gene inactivation and anemia [69, 122]. The generation of transgenic mice containing a BAC, with the Cre recombinase inserted into an approx. 200 kb DNA fragment derived from the *Epo* locus, finally allowed for the unambiguous targeting of REP cells, albeit only under hypoxic/anemic conditions [75]. To permanently label REP cells, similar transgenic mouse lines but with a Cre recombinase instead of the GFP reporter were generated and crossed with Cre-activatable reporter mice [122]. Because in this model the Cre recombinase is constitutively active, the time point of REP labelling during kidney development and episodes of tissue hypoxia remained unknown. We therefore used a similar approach, but with a conditionally regulated (i.e., tamoxifen-inducible) Cre cassette, to generate a mouse model that allowed for the exclusive targeting of currently active REP cells [50]. When crossed with a tdTomato reporter mouse, REP cells showed a bright and permanent red fluorescent signal (Fig. 3a).

In order to activate the tdTomato reporter gene, a minimal level of Cre expression is required. Under normoxic and non-anemic conditions, only very few cells were labelled with tdTomato. Far more cells were labelled following hypoxic exposure or application of a PHD inhibitor, suggesting that Cre expression levels reliably recapitulated endogenous *Epo* regulation [22, 50]. Highly sensitive ISH detected also low-level Epo mRNA expressing REP cells which were not labelled by tdTomato (Fig. 3b) possibly because Cre expression did not reach the minimal level required for reporter activation and/or due to allele-specific hypoxic *Epo* induction. Not only the number of tdTomato-positive REP cells but also the intensity of tdTomato fluorescence increased with hypoxia, which can be explained by the surprising observation that Cre sequentially recombines the tripartite transcriptional STOP cassette of the tdTomato reporter in a loxP-independent manner, allowing for a partial read-through transcription and hence a stepwise activation of the tdTomato alleles [4].

## Spatiotemporal recruitment of REP cells

Using our reporter mouse model, we could demonstrate that REP cells persist for the entire observation period of up to 32 weeks under normoxic conditions, without any Epo expression, proliferation, differentiation, or cell death. Following a second hypoxic stimulus, approx. 60% of the tagged cells can again be recruited for Epo production (Fig. 3b), independent of the duration of inactivity [22].

No labelled cells were found outside of the kidney, suggesting that the transcriptional Epo mRNA bursts are much higher in the Epo-producing cells of the kidney than those in any other organ. Intriguingly, using a liver-specific PHD inhibitor [52], a large number of hepatocytes but not REP cells could be labelled in our mouse model (manuscript in preparation), demonstrating that the observed kidney restriction in our mouse model is not due to a technical artifact, but rather represents the unexcelled physiological hypoxia sensitivity of REP cells.

Local tissue hypoxia in the REP microenvironment is likely to be caused by the massive oxygen consumption of the neighboring proximal tubule epithelial cells [25]. A pharmaceutic Epo-inducing drug would be expected to be more uniformly distributed in the kidney than tissue hypoxia. Contrary to these expectations, a PHD inhibitor (roxadustat, FG-4592) showed the same spatial distribution of tagged REP reporter cells as found after hypoxic stimulation [22], suggesting that additional factors contribute to REP recruitment by roxadustat, such as synergism with tissue hypoxia, cell–cell contacts, or paracrine mediators from the local microenvironment.

## REP cell-derived cell lines

In contrast to “ordinary” fibroblasts, isolated primary REP cells stop to proliferate and cannot be permanently cultivated in vitro [50]. Immortalization in principle would allow for indefinite proliferation of REP cells, but transgenic mice containing an *Epo*-driven SV40 large T antigen, partly even homologously recombined into the endogenous *Epo* locus, did not result in any Epo-producing cell line [69]. Cell lines derived from VHL-deficient clear cell renal cell carcinoma occasionally produce Epo, albeit in an oxygen-independent manner [95]. Cells capable of oxygen-regulated Epo expression have been reported using in vitro differentiated kidney-derived mesenchymal progenitor cells [82], and primary REP cells from kidneys of transgenic mouse lines expressing fluorescent proteins under the control of regulatory elements of the *Epo* locus (knock-in allele in a background of severe neonatal anemia) [78], or of the *Col1a1* locus [18]. Epo has also been detected in primary cells derived from 10-day-old mouse kidneys, but Epo was not hypoxically induced [1]. A similar study using 2-week-old rats resulted in CD73^+^ cells capable of hypoxic Epo induction [43]. Also human mesenchymal-like CD133^+^/CD73^+^ progenitor cells isolated from the inner medulla showed increased Epo production following hypoxic stimulation [15]. However, despite all of these approaches, there is still no kidney-derived cell line existing which would be in widespread use to investigate renal Epo regulation, similar to the human hepatoma and neuroblastoma cell lines.

Epo-driven transgenic reporter mouse models would have the potential to specifically target REP cells, but a cell line derived from constitutive Epo-Cre reporter mice lost the PDGFRβ marker as well as Epo expression [85]. Therefore, we reasoned that isolation of cells acutely tagged for an “open” *Epo* locus by conditional Cre induction may enhance the chance of obtaining functional REP cells. Following immortalization with a large T antigen, we could reproducibly generate such cell lines [46, 50], but the Epo mRNA and protein levels were still quite low when compared to the human hepatoma and neuroblastoma models. Therefore, we generated additional REP-derived (REPD) cell lines from Epo-Cre^ERT2^ reporter mice, but used a heat-sensitive SV40 large T antigen for the immortalization [4, 5]. Following incubation at the non-permissive temperature (37 °C), conditionally immortalized REPD cell lines indeed increased stem cell markers and HIF-2α mRNA expression. While HIF-2α mRNA could further be induced by a neurotrophic medium, neither HIF-2α protein nor Epo was substantially increased. Vice versa, even REP cells isolated from mouse kidneys genetically deficient for VHL in PDGFRβ-positive cells, which show very high Epo mRNA levels in vivo [12, 38], rapidly lost Epo expression during in vitro cultivation despite constitutive HIF-2α stabilization by loss of VHL (M. Fuchs and A. Kurtz, Regensburg, Germany; personal communication).

In summary, these results suggest that increased HIF-2α expression/stabilization is not sufficient to prevent the decrease in Epo expression in cultured REPD cell lines. Additional transcription factor-mediated reprogramming of REPD cells seems to be required to rescue their original identity. Of note, essential coactivators of specific HIF target gene subsets have previously been identified [80, 120]. It will be of major interest to identify such master REP differentiation regulators by single-cell analyses of isolated primary REP cells.

## Why do REP cells fail to produce Epo in chronic kidney disease?

During chronic kidney disease (CKD), REP cells lose their ability to produce Epo, resulting in renal anemia that needs to be treated with erythropoiesis-stimulating agents (ESAs) in ESRD patients. The precise reason for this REP cell failure is unknown.

Acute kidney injury is often associated with global tissue hypoxia which also plays an important role during the transition to CKD [111]. In ESRD, tubulointerstitial hypoxia is caused, among others, by capillary rarefaction and extracellular matrix expansion [27, 71]. Failure of Epo production despite tissue hypoxia is obviously not compatible with normal HIF signalling, suggesting that other mechanisms are involved, including REP cell transdifferentiation or altered environmental clues such as paracrine factors or cell–cell contacts. However, the observation that ESRD patients living at higher altitudes generally require lower doses of ESAs to maintain their hematocrit [14] sparked the idea that the oxygen sensing and HIF signalling pathway in REP cells may still be intact. A fully functional HIF pathway seems also to underlie the successful clinical application of PHD inhibitors, which depends on the presence of the diseased kidney and cannot be explained by liver-derived Epo alone [7]. How could these apparently contradictory findings be resolved?

**Fig. 4:**
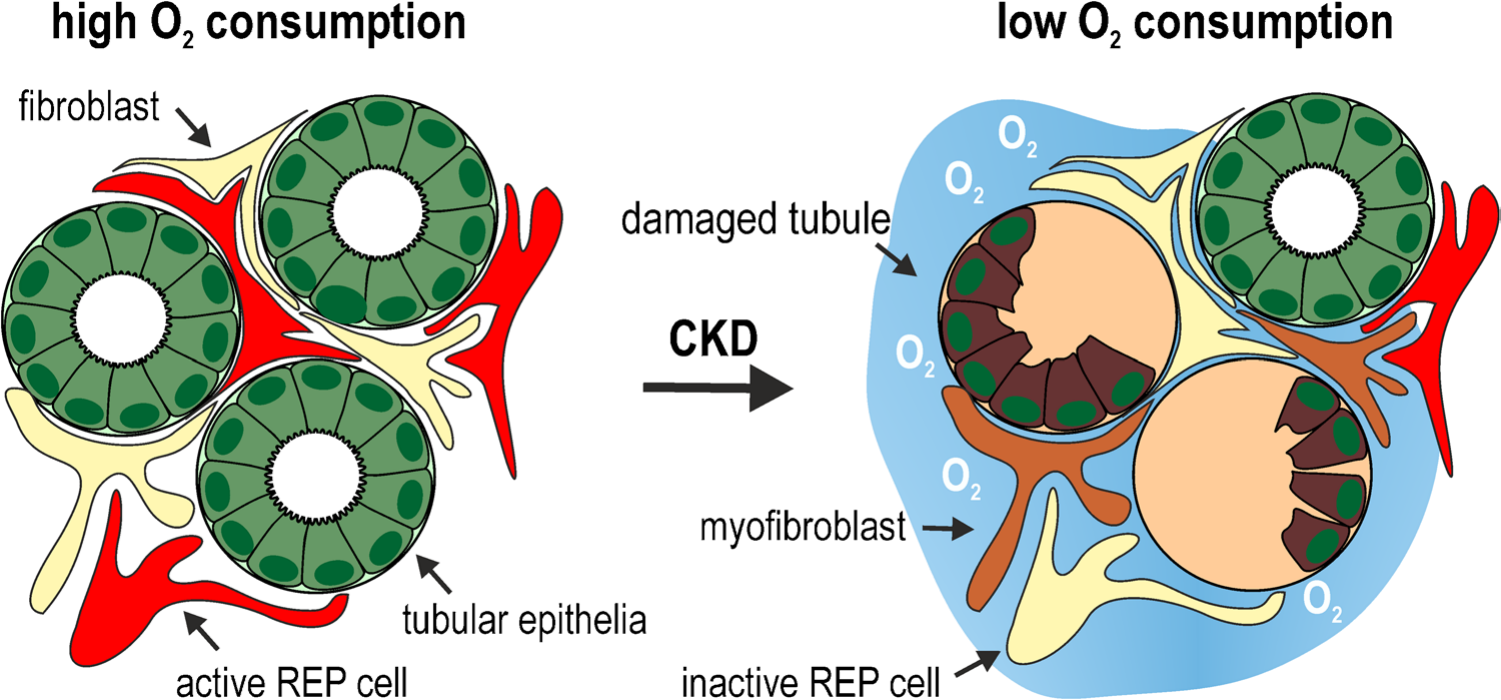
Loss of tubular function during chronic kidney disease (CKD). In this hypothetical model, renal “microenvironmental relative hyperoxia” is caused by decreased oxygen consumption of damaged tubules during the course of the disease, which leads to an attenuated hemoglobin oxygen desaturation of the capillary blood in the vicinity of the pericytic Epo-producing (REP) cells. Consequently, intracellular pO_2_ levels in REP cells exceed the normal hypoxic set point required for Epo production, leading to renal anemia

The *micro*environment of REP cells that maintained their pericytic location on capillaries that remained intact may not be affected by fibrotic tissue remodelling to a similar extent as parenchymal cells. Assuming that the oxygen content of the post-glomerular arterial blood in the vicinity of these REP cells is not affected by the renal disease, the REP cell pO_2_ is only altered by the remaining hemoglobin desaturation, caused by oxygen consumption which is well-known to predominantly depend on tubular sodium reabsorption [24, 53, 65]. Decreased proximal tubular metabolism leads to an increased cortical pO_2_ [10] and inhibits hypoxic Epo induction [25]. Due to the loss of tubular function, oxygen consumption and hence hemoglobin desaturation are likely to be decreased in the diseased kidney [27, 30, 72], possibly causing a pericytic “microenvironmental relative hyperoxia” within REP cells (Fig. 4). Vice versa, increased renal oxygen consumption by mitochondrial uncoupling led to tissue hypoxia and an increase in hemoglobin content (Epo was not measured) despite the observed nephropathy [34].

Regarding the putative changes in paracrine factors, transforming growth factor β (TGFβ) signalling has been suggested as a possible cause of suppressed or lost Epo expression in ESRD [35]. Of note, TGFβ has recently been shown to suppress HIF-2α and Epo expression [96], which may thus represent a transdifferentiation-independent direct function of the TGFβ pathway. Pro-inflammatory NF-κB signalling is also well-known to inhibit Epo expression [63], and anti-inflammatory treatment has been shown to restore Epo production in a UUO model of renal tissue remodelling [100]. Finally, epigenetic DNA modifications have been shown to silence Epo in fibrotic mouse kidneys, and DNA methyltransferase inhibitors restored Epo production [18]. However, it is currently unclear if and how PHD inhibition interferes with these processes, whereas hypoxia mimicry readily explains the reversal of a “microenvironmental relative hyperoxia” by PHD inhibitors.

Using constitutive Epo-Cre REP reporter mice, a large increase of the proportion of tagged REP cells positive for α smooth muscle actin (αSMA) has been observed [100], leading to the proposal that REP-to-myofibroblast transdifferentiation is the main cause of the loss of Epo expression during CKD [99, 110]. However, using the same CKD model, only a minor proportion of tagged REP cells expressed αSMA in our conditional Epo-Cre^ERT2^ reporter mice, and we hence concluded that Epo loss precedes transdifferentiation [22]. Because seven distinct cell clusters with increasing αSMA levels can be distinguished during myofibroblast differentiation [74], TGFβ-induced αSMA does not necessarily represent fully differentiated myofibroblasts.

## Efficient rescue of inactive REP cells by PHD inhibitors to treat renal anemia

Epo-deficient renal anemia needs to be treated by ESA injections or, more recently, by oral application of HIFα stabilizing PHD inhibitors [57]. Of the five drugs that have recently been clinically approved [45], roxadustat was the first compound authorized for the treatment of renal anemia. While the known rapid induction of Epo by roxadustat [22, 23, 48] would be consistent with a counteraction of “microenvironmental relative hyperoxia” [49, 88], it is more difficult to understand how this rapid roxadustat effect could be caused by a reversal of the REP-to-myofibroblast transdifferentiation. Furthermore, it was unclear whether inactive REP cells or other cells of the large fibroblast-like cell pool are recruited by PHD inhibition.

We therefore applied a unilateral model of renal tissue remodelling to our conditional Epo-Cre^ERT2^ reporter mice. Epo expression was completely abrogated in the damaged but not contralateral or sham-operated kidneys. Importantly, we found that roxadustat recruited previously active tagged REP cells of the damaged kidney and that roxadustat efficiently induced Epo expression in the damaged kidney to the same extent as in the (healthy) contralateral kidney [22], demonstrating that disease-inactivated REP cells can readily be recruited by hypoxia-mimicking agents.

Recently, Kobayashi et al. suggested that Epo is lost during CKD progression due to the progressive loss of areas containing functional REP cells and that phlebotomy or PHD inhibition re-induced Epo in REP cells residing in preserved areas of the otherwise damaged kidneys [54]. These preserved areas readily responded to phlebotomy but were apparently not able to prevent the Epo-dependent renal anemia that developed in these animals. Because an adenine-diet model was applied, it remains to be investigated to what degree the spatial tissue damage is comparable to the more commonly used UUO model. While the “preserved area” hypothesis also suggests regions of intact but (for unknown reasons) inactive REP cells, it may not be able to explain the PHD inhibition-mediated rescue of Epo expression in the more uniformly damaged obstructed kidney of the UUO model [22].

In conclusion, the “microenvironmental relative hyperoxia” hypothesis would explain why (i) despite global tissue hypoxia Epo is not induced, (ii) less Epo is required to treat ESRD patients at higher altitudes, and (iii) the PHD inhibitors can re-induce Epo expression as rapidly and efficiently in the diseased kidney as hypoxia does in the healthy kidney. We do not exclude that other mechanisms, such as the mentioned effects of increased TGFβ and NF-κB signalling, may contribute to the loss of Epo expression in CKD. To date, however, mechanistic explanations that would be compatible with these three observations are pending.

## How do REP cells “sense” tissue hypoxia caused by decreased blood oxygen content?

Renal arterio-venous oxygen shunts [76] and high oxygen extraction by the tubular cells ensure independence of respiratory fluctuations in the arterial pO_2_ (which are “sensed” by the carotid body). Furthermore, the stable ratio between kidney perfusion and oxygen consumption ensures blood pressure-independent oxygen sensing, explaining why systemic RBC-regulating oxygen sensing is primarily located in the kidney [116]. On the cellular level, the HIF-PHD negative feedback loop (Fig. 1) may define the hypoxic set point, allowing the cell to adapt to its microenvironmental tissue oxygen partial pressure (pO_2_) [102]. However, REP cells are pericyte-like cells, and the long processes are closely aligned to post-glomerular blood vessels in vivo [5, 98]. Likewise, the mitochondria-rich tunneling nanotubes of REPD cell lines readily aligned with vessel-like structures in vitro [5, 82]. Considering this close vicinity to blood vessels containing oxygenated arterial blood, it is challenging to understand the molecular processes that confer the unique hypoxia sensitivity to REP cells. Moreover, regarding the deep oxygen sinks of the mitochondria-rich proximal tubules of the opposite site of REP cells, there must be a high transversal oxygen flow through the pericytic REP cells, further complicating the understanding of oxygen sensing in these cells.

**Fig. 5:**
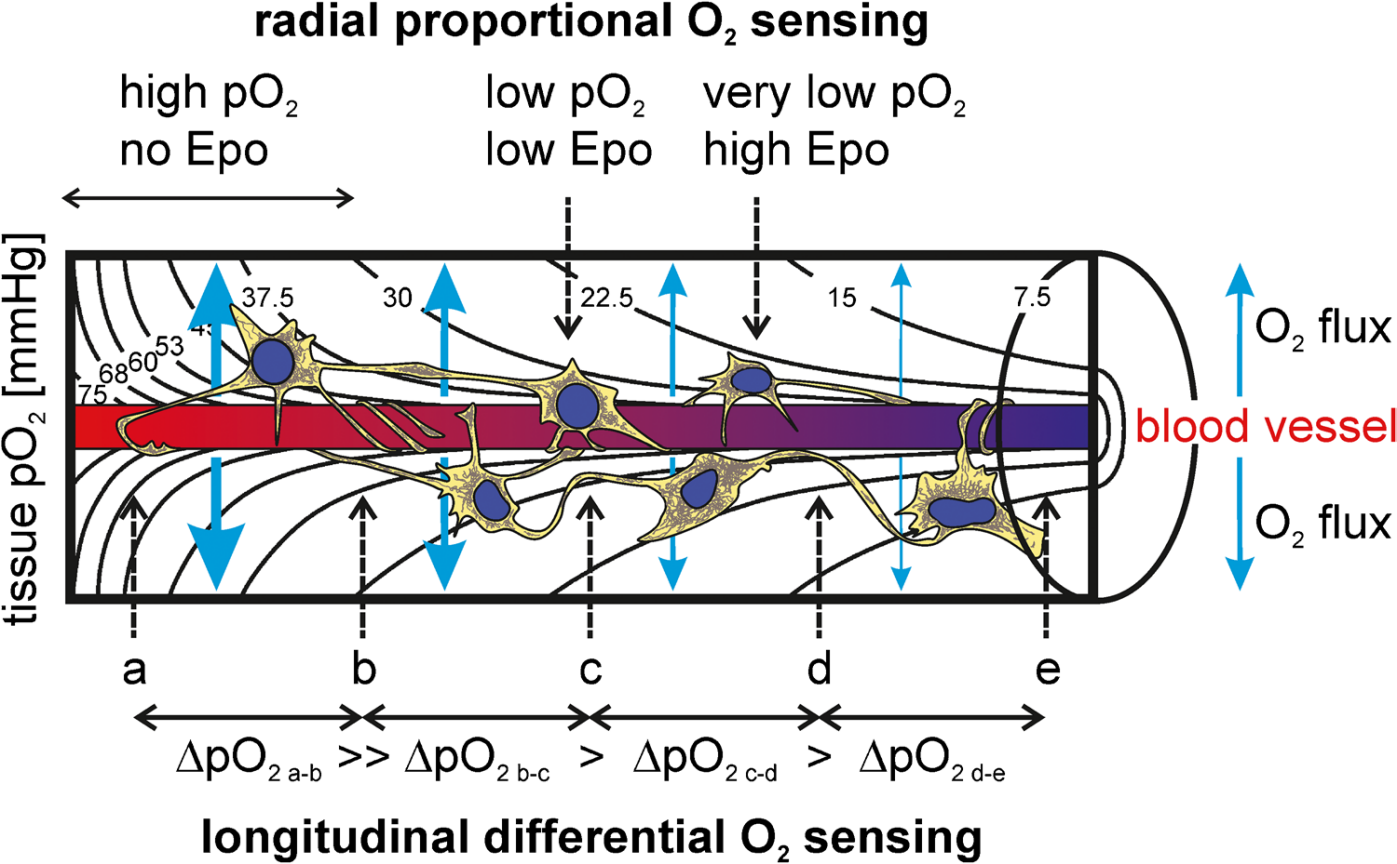
Proposed three-dimensional model for tissue oxygen partial pressure “sensing” of arterial blood oxygen content by renal Epo-producing (REP) cells. Pericytic REP cells are longitudinally aligned along post-glomerular blood vessels and are depicted within the calculated isobaric oxygen partial pressure (pO_2_) values forming the Krogh tissue cylinder [117]. Strong transversal O_2_ fluxes through REP cells, especially around the arterial end of the blood vessel, are generated by high O_2_ consumption of tubular epithelial cells located in the outer parts of the tissue cylinder (not shown). In the “radial proportional” mode (upper part), REP cells sense the mean cellular pO_2_ levels and regulate Epo in inverse proportion to this value. In the “longitudinal differential” mode (lower part), REP cells integrate the information obtained from distal pO_2_ values along the blood vessel and regulate Epo based on this differential value that provides information about the longitudinal localization. Under anemic conditions, the venous (but not the arterial) pO_2_ is lowered, and the critical tissue pO_2_ required for Epo induction is shifted towards the “arterial end” of the capillary

As illustrated by the calculated pO_2_ isobars in the Krogh tissue cylinder [117], each REP cell resides within a differently oxygenated microenvironment, depending on the longitudinal distance from the glomerulus and the radial distance from the blood vessel. It is generally assumed that Epo is produced in reverse proportion to the (radially decreasing) tissue pO_2_ (Fig. 5, upper part). While the pericytic localization of REP cells limits the radial variability of the pO_2_ microenvironment, there is still a high longitudinal variability, mainly caused by the oxygen extraction of hemoglobin by tubular oxygen consumption. For reliable hypoxia sensing, REP cells should hence obtain information about their longitudinal localization along the blood vessels. Therefore, we would like to moot a “longitudinal differential” oxygen-sensing mechanism that provides information about the localization along the blood vessel. In this model, REP cells sense the difference of the pO_2_ along the blood vessel, maybe with the help of their mitochondria-rich tunneling nanotubes which longitudinally align with blood vessels in healthy kidneys but detach and reorient towards tubules in damaged kidneys that do not produce Epo anymore [98]. Because of the sigmoidal mutual relation between hemoglobin oxygen saturation and pO_2_, the pO_2_ values drop more rapidly at the arterial end compared with the venous end of the blood vessel (assuming constant oxygen extraction by the surrounding tubular epithelia), thereby informing the REP cell about its localization along the blood vessel and/or the initial oxygen content of the post-glomerular arterial blood (Fig. 5, lower part). The high tubular oxygen extraction ensures efficient hemoglobin desaturation along short distances of the blood vessel, resulting in a high resolution of the differential tissue ΔpO_2_ values. ΔpO_2_ information may be processed by either a single REP cell or a group of REP cells connected via their tunneling nanotubes, which would be consistent with the neuronal properties of REP cells [2, 5, 75], and which would also explain the clustering that is often observed with REP cells [58].

While entirely speculative, this mechanism would be consistent with the high hypoxia sensitivity that is typical for REP cells, and the exclusive dependence on the arterial blood oxygen content. The relevant REP cell pO_2_ drops whenever the oxygen capacity of blood is lowered, by anemia as well as by hypoxemia. For instance, in an anemic patient, inspiration of 100% O_2_ would have little or no suppressive effect on renal Epo induction despite an at least fivefold increase in the arterial (but not venous) pO_2_. Vice versa, renal Epo production would be reduced in the same anemic patient if the oxygen affinity of hemoglobin would be lowered (e.g., by pharmaceutical interference with the allosteric regulation of hemoglobin O_2_ affinity by 2,3-BPG) despite unchanged arterial pO_2_.

It will be challenging to gather experimental evidence supporting this three-dimensional (3D) oxygen-sensing model. High-resolution nearest-neighborhood analyses of REP cells will be required to statistically evaluate their 3D arrangement relative to specific blood capillary sections and nephron segments. Therefore, we recently reported the combined 3D vascular and tubular imaging of whole mouse kidneys using X-ray μCT [60]. In a next step, light sheet fluorescence microscopy of the same kidney shall localize tagged REP within the μCT picture. Finally, computational modelling will be required to reveal the 3D oxygen distribution on a micrometer scale.

## Perspectives

In the absence of cell culture models that allowed for the molecular understanding of the regulatory pathways of so many other genes, most insights into renal Epo regulation have been derived from transgenic mouse models, but the nature of REP cells is still an unsolved puzzle. One possible explanation could be that multiple subtypes of fibroblast-like cells contribute to the functional REP cell pool [12]. Alternatively, REP cells represent an entirely new cell type whose currently known expression markers overlap with various other cell types. Single-cell analyses of freshly isolated REP cells bear the potential to solve this puzzle. However, due to the low expression of Epo under normoxic conditions, initial single-cell RNA sequencing (scRNAseq) data of tens of thousands kidney cells did not contain any Epo mRNA in interstitial cells [20, 26, 79, 83, 123]. Once more, tagged REP cells derived from transgenic mouse models will be instrumental in the necessary enrichment of this cell type for scRNAseq analyses.

## References

[1] Aboushwareb T, Egydio F, Straker L, Gyabaah K, Atala A, Yoo JJ (2008). Erythropoietin producing cells for potential cell therapy. World J Urol.

[2] Asada N, Takase M, Nakamura J, Oguchi A, Asada M, Suzuki N, Yamamura K, Nagoshi N, Shibata S, Rao TN, Fehling HJ, Fukatsu A, Minegishi N, Kita T, Kimura T, Okano H, Yamamoto M, Yanagita M (2011). Dysfunction of fibroblasts of extrarenal origin underlies renal fibrosis and renal anemia in mice. J Clin Invest.

[3] Auer PL, Teumer A, Schick U, O'Shaughnessy A, Lo KS, Chami N, Carlson C, de Denus S, Dubé MP, Haessler J, Jackson RD, Kooperberg C, Perreault LP, Nauck M, Peters U, Rioux JD, Schmidt F, Turcot V, Völker U, Völzke H (2014). Rare and low-frequency coding variants in CXCR2 and other genes are associated with hematological traits. Nat Genet.

[4] Bapst AM, Dahl SL, Knopfel T, Wenger RH (2020) Cre-mediated, loxP independent sequential recombination of a tripartite transcriptional stop cassette allows for partial read-through transcription. Biochim Biophys Acta Gene Regul Mech 1863:194568. 10.1016/j.bbagrm.2020.194568 10.1016/j.bbagrm.2020.194568 32344203

[5] Bapst AM, Knöpfel T, Nolan KA, Imeri F, Schuh CD, Hall AM, Guo J, Katschinski DM, Wenger RH (2022) Neurogenic and pericytic plasticity of conditionally immortalized cells derived from renal erythropoietin-producing cells. J Cell Physiol 237:2420–2433. 10.1002/jcp.30677 10.1002/jcp.30677 PMC9303970 35014036

[6] Bento C, Percy MJ, Gardie B, Maia TM, van Wijk R, Perrotta S, Della Ragione F, Almeida H, Rossi C, Girodon F, Astrom M, Neumann D, Schnittger S, Landin B, Minkov M, Randi ML, Richard S, Casadevall N, Vainchenker W, Rives S (2014). Genetic basis of congenital erythrocytosis: mutation update and online databases. Hum Mutat.

[7] Bernhardt WM, Wiesener MS, Scigalla P, Chou J, Schmieder RE, Günzler V, Eckardt KU (2010). Inhibition of prolyl hydroxylases increases erythropoietin production in ESRD. J Am Soc Nephrol.

[8] Bigham AW, Lee FS (2014). Human high-altitude adaptation: forward genetics meets the HIF pathway. Genes Dev.

[9] Ble A, Fink JC, Woodman RC, Klausner MA, Windham BG, Guralnik JM, Ferrucci L (2005). Renal function, erythropoietin, and anemia of older persons: the InCHIANTI study. Arch Intern Med.

[10] Brezis M, Agmon Y, Epstein FH (1994). Determinants of intrarenal oxygenation. I. Effects of diuretics Am J Physiol.

[11] Brines M (2010). The therapeutic potential of erythropoiesis-stimulating agents for tissue protection: a tale of two receptors. Blood Purif.

[12] Broeker KAE, Fuchs MAA, Schrankl J, Kurt B, Nolan KA, Wenger RH, Kramann R, Wagner C, Kurtz A (2020). Different subpopulations of kidney interstitial cells produce erythropoietin and factors supporting tissue oxygenation in response to hypoxia in vivo. Kidney Int.

[13] Broeker KAE, Fuchs MAA, Schrankl J, Lehrmann C, Schley G, Todorov VT, Hugo C, Wagner C, Kurtz A (2021). Prolyl-4-hydroxylases 2 and 3 control erythropoietin production in renin-expressing cells of mouse kidneys. J Physiol.

[14] Brookhart MA, Schneeweiss S, Avorn J, Bradbury BD, Rothman KJ, Fischer M, Mehta J, Winkelmayer WC (2008). The effect of altitude on dosing and response to erythropoietin in ESRD. J Am Soc Nephrol.

[15] Bussolati B, Lauritano C, Moggio A, Collino F, Mazzone M, Camussi G (2013). Renal CD133^+^/CD73^+^ progenitors produce erythropoietin under hypoxia and prolyl hydroxylase inhibition. J Am Soc Nephrol.

[16] Camps C, Petousi N, Bento C, Cario H, Copley RR, McMullin MF, van Wijk R, Ratcliffe PJ, Robbins PA, Taylor JC (2016). Gene panel sequencing improves the diagnostic work-up of patients with idiopathic erythrocytosis and identifies new mutations. Haematologica.

[17] Chambers JC, Zhang W, Li Y, Sehmi J, Wass MN, Zabaneh D, Hoggart C, Bayele H, McCarthy MI, Peltonen L, Freimer NB, Srai SK, Maxwell PH, Sternberg MJ, Ruokonen A, Abecasis G, Jarvelin MR, Scott J, Elliott P, Kooner JS (2009). Genome-wide association study identifies variants in TMPRSS6 associated with hemoglobin levels. Nat Genet.

[18] Chang YT, Yang CC, Pan SY, Chou YH, Chang FC, Lai CF, Tsai MH, Hsu HL, Lin CH, Chiang WC, Wu MS, Chu TS, Chen YM, Lin SL (2016). DNA methyltransferase inhibition restores erythropoietin production in fibrotic murine kidneys. J Clin Invest.

[19] Chen Z, Tang H, Qayyum R, Schick UM, Nalls MA, Handsaker R, Li J, Lu Y, Yanek LR, Keating B, Meng Y, van Rooij FJ, Okada Y, Kubo M, Rasmussen-Torvik L, Keller MF, Lange L, Evans M, Bottinger EP, Linderman MD (2013). Genome-wide association analysis of red blood cell traits in African Americans: the COGENT Network. Hum Mol Genet.

[20] Combes AN, Phipson B, Lawlor KT, Dorison A, Patrick R, Zappia L, Harvey RP, Oshlack A, Little MH (2019) Single cell analysis of the developing mouse kidney provides deeper insight into marker gene expression and ligand-receptor crosstalk. Development 146. 10.1242/dev.178673 10.1242/dev.178673 31118232

[21] Corre T, Ponte B, Pivin E, Pruijm M, Ackermann D, Ehret G, Spanaus K, Bochud M, Wenger RH (2021). Heritability and association with distinct genetic loci of erythropoietin levels in the general population. Haematologica.

[22] Dahl SL, Pfundstein S, Hunkeler R, Dong X, Knöpfel T, Spielmann P, Scholz CC, Nolan KA, Wenger RH (2022). Fate-mapping of erythropoietin-producing cells in mouse models of hypoxaemia and renal tissue remodelling reveals repeated recruitment and persistent functionality. Acta Physiol (Oxf).

[23] Del Balzo U, Signore PE, Walkinshaw G, Seeley TW, Brenner MC, Wang Q, Guo G, Arend MP, Flippin LA, Chow FA, Gervasi DC, Kjaergaard CH, Langsetmo I, Guenzler V, Liu DY, Klaus SJ, Lin A, Neff TB (2020). Nonclinical characterization of the hypoxia-inducible factor prolyl hydroxylase inhibitor roxadustat, a novel treatment of anemia of chronic kidney disease. J Pharmacol Exp Ther.

[24] Dizin E, Olivier V, Roth I, Sassi A, Arnoux G, Ramakrishnan S, Morel S, Kwak B, Loffing J, Hummler E, Wenger RH, Frew I, Feraille E (2021). Activation of the hypoxia-inducible factor pathway inhibits epithelial sodium channel-mediated sodium transport in collecting duct principal cells. J Am Soc Nephrol.

[25] Eckardt KU, Kurtz A, Bauer C (1989). Regulation of erythropoietin production is related to proximal tubular function. Am J Physiol.

[26] England AR, Chaney CP, Das A, Patel M, Malewska A, Armendariz D, Hon GC, Strand DW, Drake KA, Carroll TJ (2020) Identification and characterization of cellular heterogeneity within the developing renal interstitium. Development 147. 10.1242/dev.190108 10.1242/dev.190108 PMC7438011 32586976

[27] Faivre A, Scholz CC, de Seigneux S (2021). Hypoxia in chronic kidney disease: towards a paradigm shift?. Nephrol Dial Transplant.

[28] Fandrey J, Schodel J, Eckardt KU, Katschinski DM, Wenger RH (2019). Now a Nobel gas: oxygen. Eur J Physiol.

[29] Farrell JJ, Sherva RM, Chen ZY, Luo HY, Chu BF, Ha SY, Li CK, Lee AC, Li RC, Li CK, Yuen HL, So JC, Ma ES, Chan LC, Chan V, Sebastiani P, Farrer LA, Baldwin CT, Steinberg MH, Chui DH (2011). A 3-bp deletion in the *HBS1L-MYB* intergenic region on chromosome 6q23 is associated with HbF expression. Blood.

[30] Farsijani NM, Liu Q, Kobayashi H, Davidoff O, Sha F, Fandrey J, Ikizler TA, O'Connor PM, Haase VH (2016). Renal epithelium regulates erythropoiesis via HIF-dependent suppression of erythropoietin. J Clin Invest.

[31] Flashman E, Davies SL, Yeoh KK, Schofield CJ (2010). Investigating the dependence of the hypoxia-inducible factor hydroxylases (factor inhibiting HIF and prolyl hydroxylase domain 2) on ascorbate and other reducing agents. Biochem J.

[32] Franke K, Kalucka J, Mamlouk S, Singh RP, Muschter A, Weidemann A, Iyengar V, Jahn S, Wieczorek K, Geiger K, Muders M, Sykes AM, Poitz DM, Ripich T, Otto T, Bergmann S, Breier G, Baretton G, Fong GH, Greaves DR (2013). HIF-1α is a protective factor in conditional PHD2-deficient mice suffering from severe HIF-2α-induced excessive erythropoiesis. Blood.

[33] Fried W (1972). The liver as a source of extrarenal erythropoietin production. Blood.

[34] Friederich-Persson M, Thörn E, Hansell P, Nangaku M, Levin M, Palm F (2013). Kidney hypoxia, attributable to increased oxygen consumption, induces nephropathy independently of hyperglycemia and oxidative stress. Hypertension.

[35] Fuchs MAA, Broeker KAE, Schrankl J, Burzlaff N, Willam C, Wagner C, Kurtz A (2021). Inhibition of transforming growth factor β1 signaling in resident interstitial cells attenuates profibrotic gene expression and preserves erythropoietin production during experimental kidney fibrosis in mice. Kidney Int.

[36] Ganesh SK, Zakai NA, van Rooij FJ, Soranzo N, Smith AV, Nalls MA, Chen MH, Kottgen A, Glazer NL, Dehghan A, Kuhnel B, Aspelund T, Yang Q, Tanaka T, Jaffe A, Bis JC, Verwoert GC, Teumer A, Fox CS, Guralnik JM (2009). Multiple loci influence erythrocyte phenotypes in the CHARGE Consortium. Nat Genet.

[37] Gerl K, Miquerol L, Todorov VT, Hugo CP, Adams RH, Kurtz A, Kurt B (2015). Inducible glomerular erythropoietin production in the adult kidney. Kidney Int.

[38] Gerl K, Nolan KA, Karger C, Fuchs M, Wenger RH, Stolt CC, Willam C, Kurtz A, Kurt B (2016). Erythropoietin production by PDGFR-β^+^ cells. Eur J Physiol.

[39] Goch J, Birgegard G, Wikstrom B, Backman U, Wadstrom J, Danielson BG (1996). Serum erythropoietin and erythropoiesis during six years after kidney transplantation. Nephron.

[40] Goch J, Birgegard G, Wikstrom B, Tufveson G, Danielson BG (1992). Serum erythropoietin levels in the immediate kidney-posttransplant period. Nephron.

[41] Goldberg MA, Glass GA, Cunningham JM, Bunn HF (1987). The regulated expression of erythropoietin by two human hepatoma cell lines. Proc Natl Acad Sci USA.

[42] Grote Beverborg N, Verweij N, Klip IT, van der Wal HH, Voors AA, van Veldhuisen DJ, Gansevoort RT, Bakker SJ, van der Harst P, van der Meer P (2015). Erythropoietin in the general population: reference ranges and clinical, biochemical and genetic correlates. PLoS ONE.

[43] Gyabaah K, Aboushwareb T, Guimaraes Souza N, Yamaleyeva L, Varner A, Wang HJ, Atala A, Yoo JJ (2012). Controlled regulation of erythropoietin by primary cultured renal cells for renal failure induced anemia. J Urol.

[44] Haase VH (2010). Hypoxic regulation of erythropoiesis and iron metabolism. Am J Physiol Renal Physiol.

[45] Haase VH (2011). (2021) Hypoxia-inducible factor-prolyl hydroxylase inhibitors in the treatment of anemia of chronic kidney disease. Kidney Int Suppl.

[46] Hafizi R, Imeri F, Wenger RH, Huwiler A (2021) S1P stimulates erythropoietin production in mouse renal interstitial fibroblasts by S1P(1) and S1P(3) receptor activation and HIF-2α stabilization. Int J Mol Sci 22. 10.3390/ijms22179467 10.3390/ijms22179467 PMC8430949 34502385

[47] Hirano I, Suzuki N, Yamazaki S, Sekine H, Minegishi N, Shimizu R, Yamamoto M (2017). Renal anemia model mouse established by transgenic rescue with an erythropoietin gene lacking kidney-specific regulatory elements. Mol Cell Biol.

[48] Hoppe G, Yoon S, Gopalan B, Savage AR, Brown R, Case K, Vasanji A, Chan ER, Silver RB, Sears JE (2016). Comparative systems pharmacology of HIF stabilization in the prevention of retinopathy of prematurity. Proc Natl Acad Sci USA.

[49] Huang LT, Chou HC, Chen CM (2021). Roxadustat attenuates hyperoxia-induced lung injury by upregulating proangiogenic factors in newborn mice. Pediatr Neonatol.

[50] Imeri F, Nolan KA, Bapst AM, Santambrogio S, Abreu-Rodríguez I, Spielmann P, Pfundstein S, Libertini S, Crowther L, Orlando IMC, Dahl SL, Keodara A, Kuo W, Kurtcuoglu V, Scholz CC, Qi W, Hummler E, Hoogewijs D, Wenger RH (2019). Generation of renal Epo-producing cell lines by conditional gene tagging reveals rapid HIF-2 driven Epo kinetics, cell autonomous feedback regulation, and a telocyte phenotype. Kidney Int.

[51] Jelkmann W, Wiedemann G (1989). Lack of sex dependence of the serum level of immunoreactive erythropoietin in chronic anemia. Klin Wochenschr.

[52] Kato S, Ochiai N, Takano H, Io F, Takayama N, Koretsune H, Kunioka EI, Uchida S, Yamamoto K (2019). TP0463518, a novel prolyl hydroxylase inhibitor, specifically induces erythropoietin production in the liver. J Pharmacol Exp Ther.

[53] Kiil F, Aukland K, Refsum HE (1961). Renal sodium transport and oxygen consumption. Am J Physiol.

[54] Kobayashi H, Davidoff O, Pujari-Palmer S, Drevin M, Haase VH (2022) EPO synthesis induced by HIF-PHD inhibition is dependent on myofibroblast transdifferentiation and colocalizes with non-injured nephron segments in murine kidney fibrosis. Acta Physiol (Oxf):e13826. doi:10.1111/apha.13826 10.1111/apha.13826 PMC9329237 35491502

[55] Kobayashi H, Liu Q, Binns TC, Urrutia AA, Davidoff O, Kapitsinou PP, Pfaff AS, Olauson H, Wernerson A, Fogo AB, Fong GH, Gross KW, Haase VH (2016). Distinct subpopulations of FOXD1 stroma-derived cells regulate renal erythropoietin. J Clin Invest.

[56] Köchling J, Curtin PT, Madan A (1998). Regulation of human erythropoietin gene induction by upstream flanking sequences in transgenic mice. Br J Haematol.

[57] Koury MJ, Haase VH (2015). Anaemia in kidney disease: harnessing hypoxia responses for therapy. Nat Rev Nephrol.

[58] Koury ST, Koury MJ, Bondurant MC, Caro J, Graber SE (1989). Quantitation of erythropoietin-producing cells in kidneys of mice by *in situ* hybridization: correlation with hematocrit, renal erythropoietin mRNA, and serum erythropoietin concentration. Blood.

[59] Kullo IJ, Ding K, Jouni H, Smith CY, Chute CG (2010) A genome-wide association study of red blood cell traits using the electronic medical record. PLoS ONE 5. 10.1371/journal.pone.0013011 10.1371/journal.pone.0013011 PMC2946914 20927387

[60] Kuo W, Le NA, Spingler B, Wenger RH, Kipar A, Hetzel U, Schulz G, Müller B, Kurtcuoglu V (2020). Simultaneous three-dimensional vascular and tubular imaging of whole mouse kidneys with X-ray μCT. Microsc Microanal.

[61] Kurt B, Gerl K, Karger C, Schwarzensteiner I, Kurtz A (2015). Chronic hypoxia-inducible transcription factor-2 activation stably transforms juxtaglomerular renin cells into fibroblast-like cells in vivo. J Am Soc Nephrol.

[62] Kurt B, Paliege A, Willam C, Schwarzensteiner I, Schucht K, Neymeyer H, Sequeira-Lopez ML, Bachmann S, Gomez RA, Eckardt KU, Kurtz A (2013). Deletion of von Hippel-Lindau protein converts renin-producing cells into erythropoietin-producing cells. J Am Soc Nephrol.

[63] La Ferla K, Reimann C, Jelkmann W, Hellwig-Bürgel T (2002). Inhibition of erythropoietin gene expression signaling involves the transcription factors GATA-2 and NF-κB. FASEB J.

[64] Lappin TR, Lee FS (2019). Update on mutations in the HIF: EPO pathway and their role in erythrocytosis. Blood Rev.

[65] Lassen NA, Munck O, Thaysen JH (1961). Oxygen consumption and sodium reabsorption in the kidney. Acta Physiol Scand.

[66] Lee FS, Percy MJ (2011). The HIF pathway and erythrocytosis. Annu Rev Pathol.

[67] Madan A, Curtin PT (1993). A 24-base-pair sequence 3′ to the human erythropoietin gene contains a hypoxia-responsive transcriptional enhancer. Proc Natl Acad Sci USA.

[68] Madan A, Lin C, Hatch SLI, Curtin PT (1995). Regulated basal, inducible, and tissue-specific human erythropoietin gene expression in transgenic mice requires multiple *cis* DNA sequences. Blood.

[69] Maxwell PH, Osmond MK, Pugh CW, Heryet A, Nicholls LG, Tan CC, Doe BG, Ferguson DJ, Johnson MH, Ratcliffe PJ (1993). Identification of the renal erythropoietin-producing cells using transgenic mice. Kidney Int.

[70] Maxwell PH, Pugh CW, Ratcliffe PJ (1993). Inducible operation of the erythropoietin 3′ enhancer in multiple cell lines: evidence for a widespread oxygen-sensing mechanism. Proc Natl Acad Sci USA.

[71] Mimura I, Nangaku M (2010). The suffocating kidney: tubulointerstitial hypoxia in end-stage renal disease. Nat Rev Nephrol.

[72] Nolan KA, Wenger RH (2018). Source and microenvironmental regulation of erythropoietin in the kidney. Curr Opin Nephrol Hypertens.

[73] Nytko KJ, Maeda N, Schläfli P, Spielmann P, Wenger RH, Stiehl DP (2011). Vitamin C is dispensable for oxygen sensing *in vivo*. Blood.

[74] Ó hAinmhire E, Wu H, Muto Y, Donnelly EL, Machado FG, Fan LX, Chang-Panesso M, Humphreys BD,  (2019). A conditionally immortalized Gli1-positive kidney mesenchymal cell line models myofibroblast transition. Am J Physiol Renal Physiol.

[75] Obara N, Suzuki N, Kim K, Nagasawa T, Imagawa S, Yamamoto M (2008). Repression via the GATA box is essential for tissue-specific *erythropoietin* gene expression. Blood.

[76] Olgac U, Kurtcuoglu V (2015). Renal oxygenation: preglomerular vasculature is an unlikely contributor to renal oxygen shunting. Am J Physiol Renal Physiol.

[77] Orlando IMC, Lafleur VN, Storti F, Spielmann P, Crowther L, Santambrogio S, Schödel J, Hoogewijs D, Mole DR, Wenger RH (2020). Distal and proximal hypoxia response elements cooperate to regulate organ-specific erythropoietin gene expression. Haematologica.

[78] Pan X, Suzuki N, Hirano I, Yamazaki S, Minegishi N, Yamamoto M (2011). Isolation and characterization of renal erythropoietin-producing cells from genetically produced anemia mice. PLoS ONE.

[79] Park J, Shrestha R, Qiu C, Kondo A, Huang S, Werth M, Li M, Barasch J, Suszták K (2018). Single-cell transcriptomics of the mouse kidney reveals potential cellular targets of kidney disease. Science.

[80] Pawlus MR, Wang L, Ware K, Hu CJ (2012). Upstream stimulatory factor 2 and hypoxia-inducible factor 2α (HIF2α) cooperatively activate HIF2 target genes during hypoxia. Mol Cell Biol.

[81] Perrotta S, Stiehl DP, Punzo F, Scianguetta S, Borriello A, Bencivenga D, Casale M, Nobili B, Fasoli S, Balduzzi A, Cro L, Nytko KJ, Wenger RH, Della Ragione F (2013). Congenital erythrocytosis associated with gain-of-function *HIF2A* gene mutations and erythropoietin levels in the normal range. Haematologica.

[82] Plotkin MD, Goligorsky MS (2006). Mesenchymal cells from adult kidney support angiogenesis and differentiate into multiple interstitial cell types including erythropoietin-producing fibroblasts. Am J Physiol Renal Physiol.

[83] Ransick A, Lindström NO, Liu J, Zhu Q, Guo JJ, Alvarado GF, Kim AD, Black HG, Kim J, McMahon AP (2019). Single-cell profiling reveals sex, lineage, and regional diversity in the mouse kidney. Dev Cell.

[84] Ruschitzka FT, Wenger RH, Stallmach T, Quaschning T, de Wit C, Wagner K, Labugger R, Kelm M, Noll G, Rulicke T, Shaw S, Lindberg RL, Rodenwaldt B, Lutz H, Bauer C, Luscher TF, Gassmann M (2000). Nitric oxide prevents cardiovascular disease and determines survival in polyglobulic mice overexpressing erythropoietin. Proc Natl Acad Sci USA.

[85] Sato K, Hirano I, Sekine H, Miyauchi K, Nakai T, Kato K, Ito S, Yamamoto M, Suzuki N (2019). An immortalized cell line derived from renal erythropoietin-producing (REP) cells demonstrates their potential to transform into myofibroblasts. Sci Rep.

[86] Schödel J, Ratcliffe PJ (2019). Mechanisms of hypoxia signalling: new implications for nephrology. Nat Rev Nephrol.

[87] Schooley JC, Mahlmann LJ (1972). Erythropoietin production in the anephric rat. I. Relationship between nephrectomy, time of hypoxic exposure, and erythropoietin production. Blood.

[88] Sears JE, Hoppe G, Ebrahem Q, Anand-Apte B (2008). Prolyl hydroxylase inhibition during hyperoxia prevents oxygen-induced retinopathy. Proc Natl Acad Sci USA.

[89] Semenza GL (2009). Involvement of oxygen-sensing pathways in physiologic and pathologic erythropoiesis. Blood.

[90] Semenza GL, Dureza RC, Traystman MD, Gearhart JD, Antonarakis SE (1990). Human erythropoietin gene expression in transgenic mice: multiple transcription initiation sites and *cis*-acting regulatory elements. Mol Cell Biol.

[91] Semenza GL, Koury ST, Nejfelt MK, Gearhart JD, Antonarakis SE (1991). Cell-type-specific and hypoxia-inducible expression of the human erythropoietin gene in transgenic mice. Proc Natl Acad Sci USA.

[92] Semenza GL, Nejfelt MK, Chi SM, Antonarakis SE (1991). Hypoxia-inducible nuclear factors bind to an enhancer element located 3′ to the human erythropoietin gene. Proc Natl Acad Sci USA.

[93] Semenza GL, Traystman MD, Gearhart JD, Antonarakis SE (1989). Polycythemia in transgenic mice expressing the human erythropoietin gene. Proc Natl Acad Sci USA.

[94] Semenza GL, Wang GL (1992). A nuclear factor induced by hypoxia via de novo protein synthesis binds to the human erythropoietin gene enhancer at a site required for transcriptional activation. Mol Cell Biol.

[95] Sherwood JB, Shouval D (1986). Continuous production of erythropoietin by an established human renal carcinoma cell line: development of the cell line. Proc Natl Acad Sci USA.

[96] Shih HM, Pan SY, Wu CJ, Chou YH, Chen CY, Chang FC, Chen YT, Chiang WC, Tsai HC, Chen YM, Lin SL (2021). Transforming growth factor-β1 decreases erythropoietin production through repressing hypoxia-inducible factor 2α in erythropoietin-producing cells. J Biomed Sci.

[97] Soranzo N, Spector TD, Mangino M, Kuhnel B, Rendon A, Teumer A, Willenborg C, Wright B, Chen L, Li M, Salo P, Voight BF, Burns P, Laskowski RA, Xue Y, Menzel S, Altshuler D, Bradley JR, Bumpstead S, Burnett MS (2009). A genome-wide meta-analysis identifies 22 loci associated with eight hematological parameters in the HaemGen consortium. Nat Genet.

[98] Souma T, Nezu M, Nakano D, Yamazaki S, Hirano I, Sekine H, Dan T, Takeda K, Fong GH, Nishiyama A, Ito S, Miyata T, Yamamoto M, Suzuki N (2016). Erythropoietin synthesis in renal myofibroblasts is restored by activation of hypoxia signaling. J Am Soc Nephrol.

[99] Souma T, Suzuki N, Yamamoto M (2015). Renal erythropoietin-producing cells in health and disease. Front Physiol.

[100] Souma T, Yamazaki S, Moriguchi T, Suzuki N, Hirano I, Pan X, Minegishi N, Abe M, Kiyomoto H, Ito S, Yamamoto M (2013). Plasticity of renal erythropoietin-producing cells governs fibrosis. J Am Soc Nephrol.

[101] Stadhouders R, Aktuna S, Thongjuea S, Aghajanirefah A, Pourfarzad F, van Ijcken W, Lenhard B, Rooks H, Best S, Menzel S, Grosveld F, Thein SL, Soler E (2014). *HBS1L-MYB* intergenic variants modulate fetal hemoglobin via long-range *MYB* enhancers. J Clin Invest.

[102] Stiehl DP, Wirthner R, Köditz J, Spielmann P, Camenisch G, Wenger RH (2006). Increased prolyl 4-hydroxylase domain proteins compensate for decreased oxygen levels. Evidence for an autoregulatory oxygen-sensing system. J Biol Chem.

[103] Stolze I, Berchner-Pfannschmidt U, Freitag P, Wotzlaw C, Rössler J, Frede S, Acker H, Fandrey J (2002). Hypoxia-inducible erythropoietin gene expression in human neuroblastoma cells. Blood.

[104] Storti F, Santambrogio S, Crowther L, Otto T, Abreu-Rodríguez I, Kaufmann M, Hu CJ, Dame C, Fandrey J, Wenger RH, Hoogewijs D (2014). A novel distal upstream hypoxia response element regulating oxygen-dependent erythropoietin gene expression. Haematologica.

[105] Storz JF (2021). High-altitude adaptation: mechanistic insights from integrated genomics and physiology. Mol Biol Evol.

[106] Suzuki M, Yamazaki H, Mukai HY, Motohashi H, Shi L, Tanabe O, Engel JD, Yamamoto M (2013). Disruption of the Hbs1l-Myb locus causes hereditary persistence of fetal hemoglobin in a mouse model. Mol Cell Biol.

[107] Suzuki N, Obara N, Pan X, Watanabe M, Jishage K, Minegishi N, Yamamoto M (2011). Specific contribution of the erythropoietin gene 3′ enhancer to hepatic erythropoiesis after late embryonic stages. Mol Cell Biol.

[108] Suzuki N, Obara N, Yamamoto M (2007). Use of gene-manipulated mice in the study of erythropoietin gene expression. Methods Enzymol.

[109] Suzuki N, Ohneda O, Takahashi S, Higuchi M, Mukai HY, Nakahata T, Imagawa S, Yamamoto M (2002). Erythroid-specific expression of the erythropoietin receptor rescued its null mutant mice from lethality. Blood.

[110] Suzuki N, Yamamoto M (2016). Roles of renal erythropoietin-producing (REP) cells in the maintenance of systemic oxygen homeostasis. Eur J Physiol.

[111] Tanaka S, Tanaka T, Nangaku M (2014). Hypoxia as a key player in the AKI-to-CKD transition. Am J Physiol Renal Physiol.

[112] Taylor JC, Martin HC, Lise S, Broxholme J, Cazier JB, Rimmer A, Kanapin A, Lunter G, Fiddy S, Allan C, Aricescu AR, Attar M, Babbs C, Becq J, Beeson D, Bento C, Bignell P, Blair E, Buckle VJ, Bull K (2015). Factors influencing success of clinical genome sequencing across a broad spectrum of disorders. Nat Genet.

[113] van der Harst P, Zhang W, Mateo Leach I, Rendon A, Verweij N, Sehmi J, Paul DS, Elling U, Allayee H, Li X, Radhakrishnan A, Tan ST, Voss K, Weichenberger CX, Albers CA, Al-Hussani A, Asselbergs FW, Ciullo M, Danjou F, Dina C (2012). Seventy-five genetic loci influencing the human red blood cell. Nature.

[114] Volkova YL, Pickel C, Jucht AE, Wenger RH, Scholz CC (2022). The asparagine hydroxylase FIH - a unique oxygen sensor. Antioxid Redox Signal.

[115] Wang Y, Nudel R, Benros ME, Skogstrand K, Fishilevich S, Lancet D, Sun J, Hougaard DM, Andreassen OA, Mortensen PB, Buil A, Hansen TF, Thompson WK, Werge T (2020). Genome-wide association study identifies 16 genomic regions associated with circulating cytokines at birth. PLoS Genet.

[116] Wenger RH, Hoogewijs D (2010). Regulated oxygen sensing by protein hydroxylation in renal erythropoietin-producing cells. Am J Physiol Renal Physiol.

[117] Wenger RH, Kurtcuoglu V, Scholz CC, Marti HH, Hoogewijs D (2015). Frequently asked questions in hypoxia research. Hypoxia.

[118] Wenger RH, Kurtz A (2011). Erythropoietin Compr Physiol.

[119] Wenger RH, Stiehl DP, Camenisch G (2005) Integration of oxygen signaling at the consensus HRE. Sci STKE 2005:re12. doi:10.1126/stke.3062005re12 10.1126/stke.3062005re12 16234508

[120] Wollenick K, Hu J, Kristiansen G, Schraml P, Rehrauer H, Berchner-Pfannschmidt U, Fandrey J, Wenger RH, Stiehl DP (2012). Synthetic transactivation screening reveals ETV4 as broad coactivator of hypoxia-inducible factor signaling. Nucl Acids Res.

[121] Wu H, Liu X, Jaenisch R, Lodish HF (1995). Generation of committed erythroid BFU-E and CFU-E progenitors does not require erythropoietin or the erythropoietin receptor. Cell.

[122] Yamazaki S, Souma T, Hirano I, Pan X, Minegishi N, Suzuki N, Yamamoto M (2013). A mouse model of adult-onset anaemia due to erythropoietin deficiency. Nat Commun.

[123] Young MD, Mitchell TJ, Vieira Braga FA, Tran MGB, Stewart BJ, Ferdinand JR, Collord G, Botting RA, Popescu DM, Loudon KW, Vento-Tormo R, Stephenson E, Cagan A, Farndon SJ, Del Castillo V-H, Guzzo C, Richoz N, Mamanova L, Aho T, Armitage JN (2018). Single-cell transcriptomes from human kidneys reveal the cellular identity of renal tumors. Science.

[124] Zhang N, Fu Z, Linke S, Chicher J, Gorman JJ, Visk D, Haddad GG, Poellinger L, Peet DJ, Powell F, Johnson RS (2010). The asparaginyl hydroxylase factor inhibiting HIF-1α is an essential regulator of metabolism. Cell Metab.

[125] Zmajkovic J, Lundberg P, Nienhold R, Torgersen ML, Sundan A, Waage A, Skoda RC (2018). A gain-of-function mutation in EPO in familial erythrocytosis. N Engl J Med.

